# Salivary Gland Diseases: A Retrospective Clinicopathological Study of 159 Cases

**DOI:** 10.7759/cureus.29589

**Published:** 2022-09-26

**Authors:** Afrah A Aldelaimi, Hamid H Enezei, Tahrir N Aldelaimi, Khalil A Mohammed, Raid M Al-Ani

**Affiliations:** 1 Department of Oral Diagnosis, University of Anbar, College of Dentistry, Ramadi, IRQ; 2 Department of Oral and Maxillofacial Surgery, University of Anbar, College of Dentistry, Ramadi, IRQ; 3 Department of Oral and Maxillofacial Surgery, University of Anbar, College of Dentistry, Ramadi Teaching Hospital, Ramadi, IRQ; 4 Department of Pathology, Ministry of Health, Anbar Health Directorate, Ramadi Teaching Hospital, Ramadi, IRQ; 5 Department of Otolaryngology, University of Anbar, College of Medicine, Ramadi, IRQ

**Keywords:** oral medicine, maxillofacial surgery, maxillofacial pathology, salivary gland pathology, salivary gland diseases

## Abstract

Background

Salivary gland diseases include a variety of conditions (inflammatory, immunological, infectious, or neoplastic pathologies). Salivary gland diseases hold the interest of clinicians and pathologists due to their varied clinical presentation and histological diversity. In this study, we aimed to assess the various aspects of clinical and pathological characteristics of salivary gland diseases.

Methodology

We reviewed the records of patients with various salivary gland diseases at Ramadi Teaching Hospital, Rashid Hospital, Razi Hospital, and Zuhur Hospital in Iraq. The study covered the years 2010 to 2021.

Results

Of 159 patients, there were 61.64% female patients. The age group most affected was 51-60 years (26.4%). The most involved salivary gland was the parotid (44.65%). Swelling was seen in 74% of the patients. Obstructive lesions were seen in 52.2% of patients. Obstructive pathologies occurred exclusively in the age group 51-60 years, infective cases involved the age group 71-80 years (64.3%), and tumors affected the age group 41-50 years (77.4%). Women were affected more than men by all pathologies. The parotid gland was mostly affected by tumors (32/71), while other glands were mostly affected by obstructive lesions (17/18). A significant association was found between salivary gland pathologies with age and the affected gland. The most common clinical entity of the obstructive lesions was xerostomia (20.1%). While pleomorphic adenoma was the most common tumor (n = 40/50). The most common cause of xerostomia was smoking (31.2%) and the least cause was antidepressants (9.4%).

Conclusions

Salivary gland diseases were mostly seen in women and in the age group 51-60 years. Parotid was the most involved gland. A three-quarter of the cases presented with swelling and obstructive pathologies comprise above 50% of causes. The age and the involved gland can determine the type of salivary gland diseases. Xerostomia was the common clinical entity of obstructive pathologies. The most common tumor was pleomorphic adenoma and the most common cause for xerostomia was smoking.

## Introduction

Salivary glands are the exocrine organs for saliva production and secretion that consist of three pairs of major salivary glands (parotid, submandibular, and sublingual glands) as well as hundreds of minor salivary glands distributed in the lining mucosa of the upper aerodigestive tract [1]. Saliva is a highly complex mixture of water, organic, and non-organic materials. It aids in the lubrication of the oral cavity, mastication, swallowing, and protection of the mouth and teeth [2].

Salivary glands comprise an enormous spectrum of non-neoplastic (autoimmune, inflammatory, infections) and neoplastic disorders (benign and malignant). Their diagnosis depends on histopathological and clinical aspects and sometimes on complementary analyses. However, in some cases, the clinical diagnostic impression is enough to establish a definitive diagnosis [3].

Salivary gland tumors are categorized into malignant and benign tumors. The majority of tumors are located in the parotid gland, most of them are benign, and most of the benign tumors are pleomorphic adenomas [4]. In 2005, the World Health Organization (WHO) recognized 24 malignant tumors, including mucoepidermoid carcinoma, acinic cell carcinoma, adenoid cystic carcinoma, carcinoma ex-pleomorphic adenoma, and adenocarcinoma [5]. Minor salivary gland tumors are mainly located in the palate, tongue, lips, pharynx, buccal mucosa, paranasal sinuses, and larynx. These tumors have a female preponderance and account for about 9-25% of all salivary gland tumors. Moreover, most of the minor salivary gland tumors are malignant [6].

Sialolithiasis is one of the most common benign conditions that affect the salivary glands. It is caused by the formation of sialolith/calculus within their parenchyma or ductal system. Sialoliths are composed of several ratios of organic and inorganic substances. However, their mechanism of formation remains incompletely understood. Some studies have shown that sialolithiasis is more frequently located in major salivary glands, particularly in the submandibular and parotid glands. This is likely due to the long and tortuous path of the major duct as well as the nature and consistency of the submandibular gland saliva, which is thicker in consistency, rich in phosphorous, and has a high pH that is conducive to sialolith formation [7].

Myoepithelial sialadenitis (MESA) is featured by the formation of epi-myoepithelial islands with chronic lymphocytic infiltration in the salivary parenchyma often with glandular atrophy. A recent study reported a case of MESA in which the excised parotid gland tissue had several calcified laminated calculi scattered among dilated ducts [8].

A relatively low number of patients with salivary gland diseases present in our hospitals. Knowing the various demographic, clinical, and pathological aspects of these disorders will provide an impetus for better management of affected subjects. Hence, we conducted this study to assess the different aspects of the clinical and pathological characteristics of salivary gland diseases among patients in Iraq.

## Materials and methods

In this retrospective study, 159 patients with salivary gland diseases were enrolled. The study was conducted at Ramadi Teaching Hospital, Rashid Hospital, Razi Hospital, and Zuhur Hospital in Iraq. This study was conducted over a period of 12 years (from 2010 to 2021). Owing to the retrospective nature of the study, ethical approval was waived. Patients aged ≥18 years of both sexes diagnosed with salivary gland diseases were included in this study. Patients aged <18 years, those with congenital malformations of salivary glands, and those with incomplete data on medical records were excluded. Data were taken from each participant regarding their age, gender, type and location of disease, and clinical features.

Intraoral and extraoral clinical examinations were performed through both soft and bony tissue gentle digital palpation, along with a thorough inspection for proper evaluation and assessment of lesions regarding size, shape, consistency, location, surface texture, the color of the overlying tissue, fluctuation, and loco-regional lymphadenopathy.

**Figure 1:**
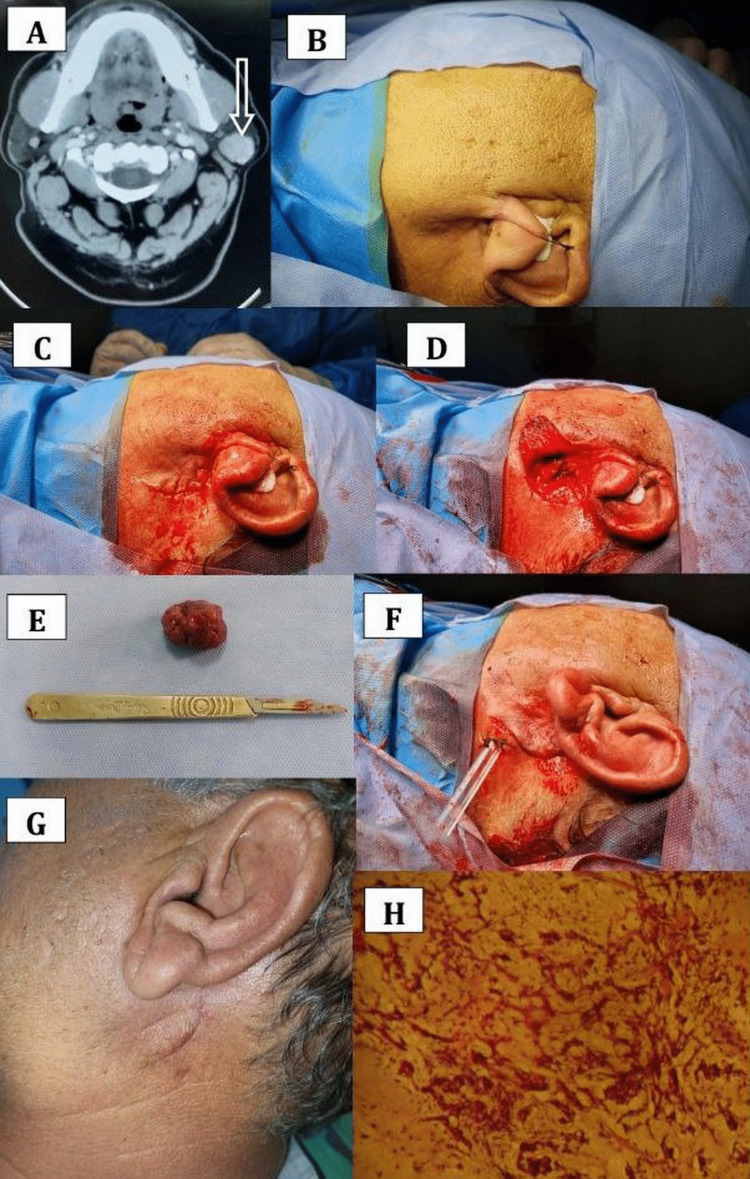
(A) Axial CT revealing (arrow) a mass 4 × 2.5 cm in the left parotid gland. (B) Preoperative view and site prepared for surgery. (C) Incision. (D) Superficial parotidectomy done with preservation of the facial nerve. (E) Surgical specimen of the tumor. (F) Wound closure with drain placement. (G) Postoperative view four weeks after the surgery. (H) Histopathological confirmation (H&E stain, 100×) of pleomorphic adenoma. CT: computed tomography; H&E: hematoxylin and eosin

The radiographical examination was conducted using conventional radiography, computed tomography (CT), ultrasonic examination with or without fine-needle aspiration cytology (FNAC), and magnetic resonance imaging (MRI). These radiological as well as medical laboratory investigations were requested as needed depending on the medical history, clinical entity, and differential diagnosis of the case. In case of a tumor or highly suspicious mass, an incisional or excisional biopsy was performed to reach the final diagnosis of the lesion. Figure 1 is an example of a patient seen during the study period.

The patients were divided into three categories according to the pathologies, namely, obstructive, infective, and tumors. A comparison among these groups regarding the age, gender, and type of glands was done.

The data were processed SPSS version 25 for windows (IBM Corp., Armonk, NY, USA). The mean ± standard deviation (SD) or median ± interquartile range (IQR) were calculated. The chi-square test was used to compare categorical variables. A p-value of less than 0.05 was considered a statistically significant difference.

## Results

Out of 175 patients with salivary gland diseases, 159 fulfilled the inclusion criteria and were enrolled in the study. The age of the patients ranged from 21 to 80 years (mean = 55.77 ± 14.302), while the median and mode were 57 (IQR = 57) and 42 years, respectively. The age group most affected was 51-60 years (n = 24, 26.4%) and the least affected was 21-30 years (n = 7, 4.4%). There were 98 (61.64%) females, with a male-to-female ratio was 1:1.6 (Table 1).

**Table 1:** Demographic characteristics of the 159 patients with salivary gland diseases.

Variable	Percent	Frequency
Age group (years)		
21–30	4.4	7
31–40	9.4	15
41–50	19.5	31
51–60	26.4	42
61–70	22.6	36
71–80	17.6	28
Total	100	159
Gender		
Male	38.36	61
Female	61.64	98
Total	100	159

**Figure 2:**
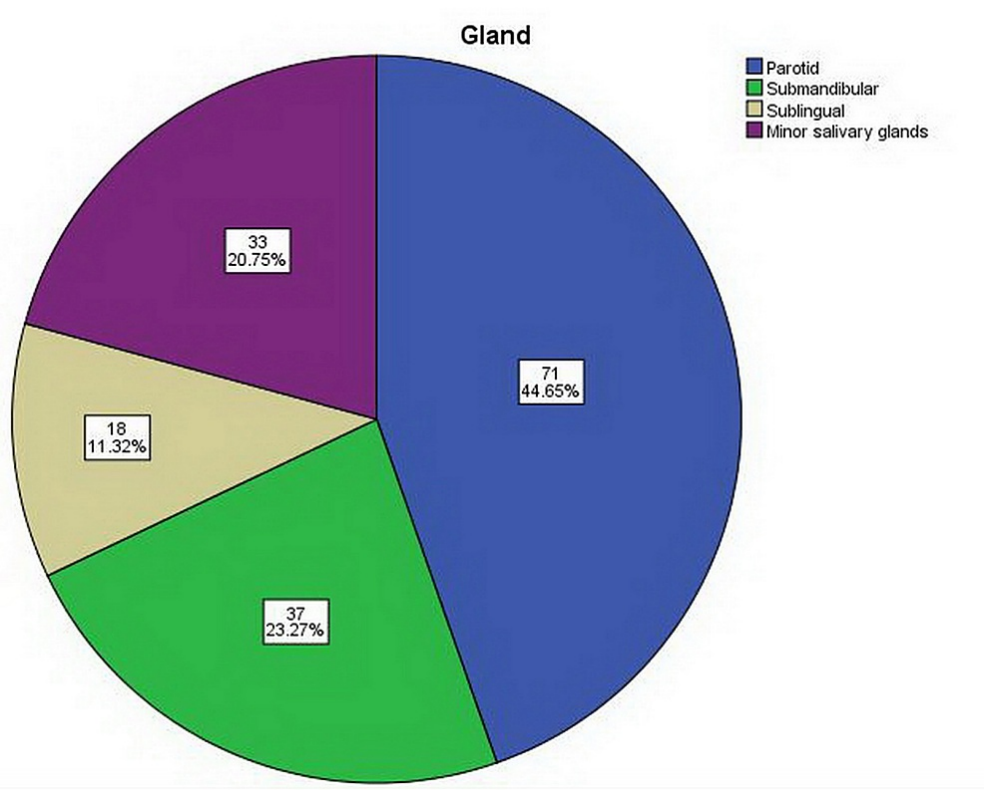
Presentation of salivary gland diseases.

The parotid gland was the most affected (n = 71, 44.65%) by diseases, while the least was the sublingual gland (n = 18, 11.32%), as shown in Figure 2.

Obstructive lesions were the most common salivary gland abnormalities (n = 83, 52.2%), while the least was pathology infection of the salivary glands (n = 26, 0.16%). Obstructive pathologies occurred exclusively in the age group 51-60 years (n = 42, 100%). Most of the infective cases involved the age group 71-80 years (n = 18, 64.3%). In the age group 41-50, tumors were the most common pathology (n = 24, 77.4%). There was a highly statistically significant difference between age and clinical entity of salivary gland diseases (p = 0.000) (Table 2).

**Table 2:** Correlation of the clinical entity with age (p = 0.000).

Age group (years)	Clinical entity			
	Obstructive, N (%)	Infection, N (%)	Tumor, N (%)	Total, N (%)
21–30	7 (100%)	0 (0%)	0 (0%)	7 (100%)
31–40	4 (26.7%)	0 (0%)	11 (73.3%)	15 (100%)
41–50	7 (22.6%)	0 (0%)	24 (77.4%)	31 (100%)
51–60	42 (100%)	0 (0%)	0 (0%)	42 (100%)
61–70	23 (63.9%)	8 (22.2%)	5 (13.9%)	36 (100%)
71–80	0 (0%)	18 (64.3%)	10 (35.7%)	28 (100%)
Total	83 (52.2%)	26 (16.4%)	50 (31.4%)	159 (100%)

**Figure 3:**
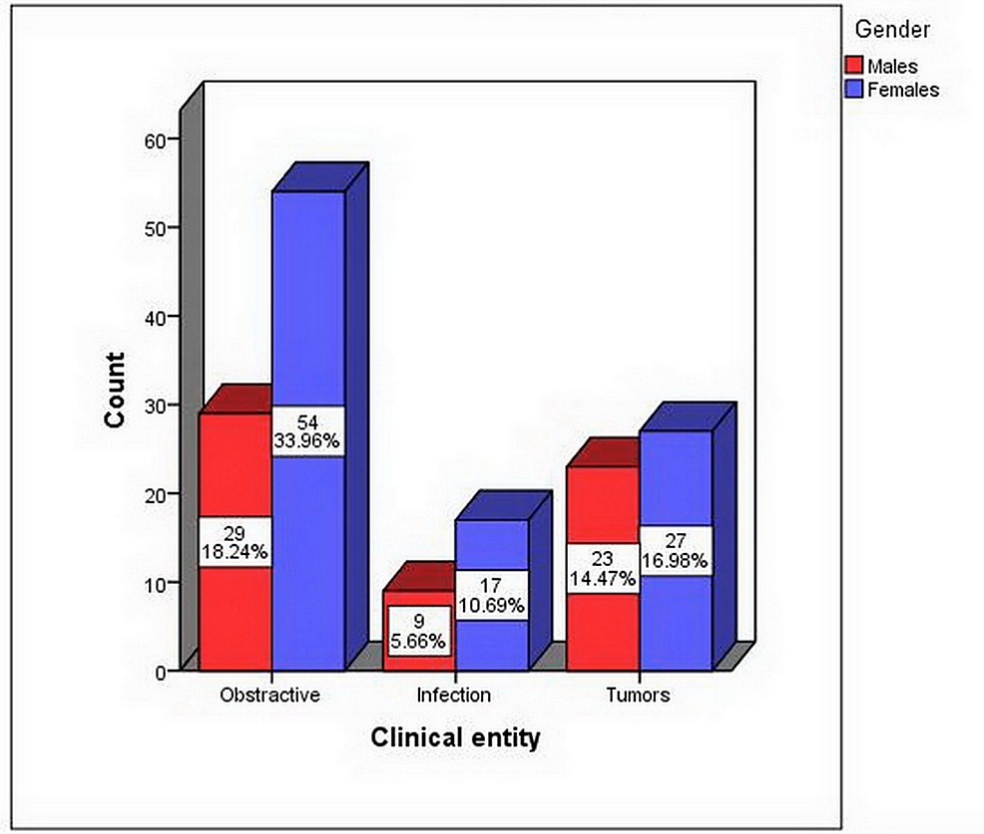
Relationship between the clinical entity and gender (p = 0.407).

Females were affected more than males by all pathologies in the following percentages: 33.96% for obstructive lesions, 16.98% for tumors, and 10.69% for infections. However, there was no statistically significant difference between gender and clinical entity of salivary gland diseases (p = 0.407) (Figure 3).

The parotid gland was mostly affected by tumors (n = 32/71), the submandibular gland by obstructive pathology (15/37), the sublingual gland by obstructive lesions (17/18), and the minor salivary gland by obstructive diseases (17/33). There was a highly statistically significant difference between the salivary gland type and clinical entity of salivary gland diseases (p = 0.000) (Table 3).

**Table 3:** Relationship between the clinical entities and the site (p = 0.000)

Salivary gland	Clinical entity			
	Obstructive, N (%)	Infection, N (%)	Tumor, N (%)	Total, N (%)
Parotid	28 (39.4%)	11 (15.50%)	32 (45.1%)	71 (100%)
Submandibular	21 (56.8%)	1 (2.7%)	15 (40.5%)	37 (100%)
Sublingual	17 (94.4%)	1 (5.6%)	0 (0%)	18 (100%)
Minor salivary glands	17 (51.5%)	13 (39.4%)	3 (9.1%)	33 (100%)
Total	83 (52.2%)	26 (16.4%)	50 (31.4%)	159 (100%)

The symptoms, in descending order, were swelling (n = 117, 74%), pain (n = 108, 68%), fever (n = 92, 58%), redness of region (n = 67, 42%), paresthesia (n = 51, 32%), xerostomia (n = 32, 20.1%), and facial nerve weakness (n = 3, 2%).

The most common clinical entity of the obstructive type was xerostomia (n = 32, 20.1%). Besides, pleomorphic adenoma was the most common tumor (n = 40, 25.2%) (Table 4).

**Table 4:** Frequency of various clinical entities.

Clinical entity		Frequency	Percent
Obstructive	Xerostomia	32	20.1%
	Sialolithiasis	21	13.2%
	Mucocele	13	8.2%
	Sialorrhea	11	6.9%
	Ranula	6	3.8%
Infection	Sialadenitis	26	16.4%
Tumor	Pleomorphic adenoma	40	25.2%
	Warthins tumor	7	4.4%
	Mucoepidermoid carcinoma	3	1.8%
Total		159	100%

The most common cause of xerostomia was smoking (n = 10, 31.2%), and the least common cause was antidepressants (n = 3, 9.4%). However, there was no statistically significant difference among the causes of xerostomia (p = 0.319) (Table 5).

**Table 5:** Causes of xerostomia in 32 patients with salivary gland diseases (p = 0.319).

Causes of xerostomia	Percent	Frequency
Smoking	31.2%	10
Antacid	18.8%	6
Antihypertensive	15.6%	5
Hypoglycemic	12.5%	4
Anti-inflammatory	12.5%	4
Antidepressants	9.4%	3
Total	100%	32

## Discussion

Various abnormalities affect the salivary glands ranging from a simple infection to aggressive tumors. Infections, sialolithiasis, and mucoceles are the most encountered pathologies of these glands [9]. However, both benign and malignant tumors also affect these glands. The majority are benign tumors, and the most common benign tumor is pleomorphic adenoma. This study reported that salivary gland disorders affect patients of any age with a female predominance. The most affected gland was the parotid. Obstructive pathologies such as sialolithiasis were the most commonly encountered clinical entity. Additionally, the study showed that there were highly statistically significant differences between salivary gland pathologies and the affected gland and the age of the patients.

Although viral infections of the salivary glands such as mumps are common in children, other salivary gland disorders are rare [10]. Gellrich et al. reported 146 pediatric patients with salivary gland disorders over a study period of 15 years [10]. Another study from Italy reported 99 children with salivary gland disorders over 20 years [11]. The majority of the studied patients were in the sixth decade of life which was similar to previous investigations [12]. In this study, obstructive salivary gland disorders had a predilection for the age group 51-60 years, which is consistent with a previous study [3] while disagreeing with another study [13]. Tumors mostly affected the age group 31-40 years, which was similar to some previous investigations [14,15] but disagreed with the findings of the study by Gireesh [16]. Further, infections were mostly seen in the age group 71-80 years, which was in disagreement with a previous investigation [3]. Besides, there was a significant association between the age of the patients and the type of lesion.

This study showed that obstructive pathologies mostly affected the parotid and submandibular glands. This confirmed the hypothesized fulfillment of the diagnostic criteria of sialolithiasis which is assumed to be formed by an initial central core of organic material which grows progressively via layered deposition of organic and inorganic materials, in addition to the mitochondria and lysosomal bodies originating from the ductal system, as well as a ductal course through oral muscle playing a role in the etiology of salivary gland stones [17]. Some studies have demonstrated that the submandibular and parotid glands are more frequently affected by sialolithiasis when compared with minor salivary glands [18,19].

According to the WHO, salivary gland diseases affect females more than males, although some differences can be found when analyzing specific tumor types. In this study, the male-to-female ratio was 1:1.6, which was consistent with the majority of studies [3,12].

Our study reported that the parotid gland is by far the most commonly affected location, with 45% of all primary salivary gland pathologies occurring at this site, followed by minor salivary glands (located in the palate, lips, etc.) in about 21%, and the submandibular gland in 23%, which was similar to previous studies [20-23].

There is a wide range of clinical features that depends on each salivary gland disorder, for example, sudden onset of pain and swelling in bacterial infections, recurrent episodes of pain and swelling often aggravated with meals, and recurrent infections in obstructive pathology such as calculus or stricture, and painless, firm, slow-growing mass in the case of tumor [1]. Therefore, swelling is the most common presentation of these disorders, as shown in this study.

Our most common finding in the clinical records was the diagnosis of xerostomia. Xerostomia can be the result of systemic diseases of endocrine, autoimmune, infectious, and granulomatous type among others, in addition to local factors such as the use of certain medications, radiation therapy of the head and neck, and some lifestyle habits such as smoking. Because there is a suggestion of the association between xerostomia and medications used to treat conditions in almost all systems of the body, some medications alone may not be enough to produce xerostomia but consuming them in combination with others may result in drug interactions and subsequent xerostomia, with a significant association between the use of antidepressants, antihypertensives, antihistamines as well as smoking with xerostomia [7,20,24,25]. The current study did not show a significant association among various causes of xerostomia (p > 0.05).

The study findings were consistent with the majority of the research groups which observed that the parotid gland is the most vulnerable to developing neoplastic lesions [3,26,27]. In this study, the benign neoplasm was present in a higher percentage than malignant cases which is consistent with other clinical observations. Moreover, the incidence of pleomorphic adenoma was higher which is consistent with the reported literature [3,27-29].

Our study included only hospitalized patients resulting in a relatively small sample size in comparison with other studies [12,22,23], which is a shortcoming of the study. The retrospective nature of the current study is another limitation of the study.

## Conclusions

Salivary gland diseases were mostly seen in women and the age group 51-60 years. Parotid was the most involved gland. Three-quarters of the cases presented with swelling and obstructive pathologies which comprised above 50% of causes. The age and the involved gland can determine the type of salivary gland diseases. However, gender did not determine the pathological lesion. Xerostomia was the common clinical entity of obstructive pathologies. Pleomorphic adenoma was the most common tumor. Smoking was the most common cause of xerostomia.
